# Giant quantum Hall plateaus generated by charge transfer in epitaxial graphene

**DOI:** 10.1038/srep30296

**Published:** 2016-07-26

**Authors:** J. A. Alexander-Webber, J. Huang, D. K. Maude, T. J. B. M. Janssen, A. Tzalenchuk, V. Antonov, T. Yager, S. Lara-Avila, S. Kubatkin, R. Yakimova, R. J. Nicholas

**Affiliations:** 1 Department of Physics, University of Oxford, Clarendon Laboratory, Parks Road, Oxford OX1 3PU, United Kingdom; 2 Laboratoire National des Champs Magnétiques Intenses, CNRS-UGA-UPS-INSA, 143, avenue de Rangueil, 31400 Toulouse, France; 3 National Physical Laboratory, Hampton Road, Teddington TW11 0LW, United Kingdom; 4 Department of Physics, Royal Holloway, University of London, Egham TW20 0EX, United Kingdom; 5 Department of Microtechnology and Nanoscience, Chalmers University of Technology, S-412 96 Göteborg, Sweden; 6 Department of Physics, Chemistry and Biology (IFM), Linköping University, S-581 83 Linköping, Sweden

## Abstract

Epitaxial graphene has proven itself to be the best candidate for quantum electrical resistance standards due to its wide quantum Hall plateaus with exceptionally high breakdown currents. However one key underlying mechanism, a magnetic field dependent charge transfer process, is yet to be fully understood. Here we report measurements of the quantum Hall effect in epitaxial graphene showing the widest quantum Hall plateau observed to date extending over 50 T, attributed to an almost linear increase in carrier density with magnetic field. This behaviour is strong evidence for field dependent charge transfer from charge reservoirs with exceptionally high densities of states in close proximity to the graphene. Using a realistic framework of broadened Landau levels we model the densities of donor states and predict the field dependence of charge transfer in excellent agreement with experimental results, thus providing a guide towards engineering epitaxial graphene for applications such as quantum metrology.

The quantum Hall effect (QHE)^1^, defined by a vanishing longitudinal resistivity *ρ*_*xx*_ = 0 and a quantised Hall resistance *ρ*_*xy*_ = *h*/*νe*^2^ for *ν* = integer, has long been used in metrology as a quantum electrical resistance standard^2^. However the traditional quantum resistance standard, based on GaAs, requires very low temperatures and high magnetic fields to achieve suitable levels of precision. Recently we have shown that graphene, grown epitaxially on SiC, can maintain the quantum Hall state up to critical current densities *j*_*c*_ which can be more than a factor 30 larger than previously observed in other material systems^3^. Furthermore, these high values of *j*_*c*_ were also observed to persist up to significantly higher critical temperatures *T*_*c*_ when compared to GaAs for a given magnetic field^4^, making graphene the best candidate for the next generation of quantum resistance metrology applications^5^,^6^,^7^. This improved performance of graphene is in part due to the large cyclotron energy gaps arising from the high electron velocity at the Dirac point^8^, and is also due to the high electron-phonon energy relaxation rates, an order of magnitude faster than in GaAs heterostructures, which play an important role in determining the high current breakdown of the QHE^9^,^10^.

In epitaxial graphene grown on the Si-terminated face of SiC, charge transfer from the underlying substrate to the graphene leads to a strongly magnetic field dependent carrier density and an exceptionally wide quantised *ν* = 2 plateau^11^,^12^. The mechanisms which underlie this behaviour need to be understood if epitaxial graphene is to live up to its metrological potential. However, due to this charge transfer process and the high magnetic fields to which *ν* = 2 is extended it is very difficult to experimentally observe the full extent of the *ν* = 2 plateau^3^,^6^,^12^,^13^,^14^,^15^,^16^,^17^,^18^,^19^,^20^,^21^. Initial theoretical descriptions of this phenomena are based on charge transfer from the underlying substrate, due to the large quantum capacitance in the system, into the *N* = 0 Landau level in graphene which has fixed zero energy independent of magnetic field^11^,^12^. These predict a linear magnetic field dependence of the carrier density *n* in the *ν* = 2 plateau such that the *N* = 0 Landau level remains exactly filled over a large range of magnetic fields resulting in the exceptionally wide plateau and high critical current densities. In these models the linear magnetic field dependence of *n* is predicted to saturate at high magnetic fields. However, further work has suggested that the carrier density continues to increase to much higher magnetic fields^3^. The assumptions made in early theoretical models include unbroadened *δ*-function Landau levels in graphene and constant densities of states within the charge reservoirs. Experimental data in which the full extent of the magnetic field dependence can be measured is therefore crucial in obtaining a complete understanding of this process which underpins the benefits of graphene in quantum metrology. Using a combination of pulsed high magnetic fields up to 57 T and very low carrier density samples we present here the first detailed study of the full magnetic field dependence of charge transfer in epitaxial graphene through a *ν* = 2 plateau which remarkably extends from *B* = 3 T to over 50 T. We show that by using the occupancy dependence of the quantum Hall breakdown current we can demonstrate that the carrier density increases almost linearly throughout the *ν* = 2 plateau. This method is validated using a fit to the temperature dependence of the magneto-conductivity which provides a direct measure of the position of the Fermi energy with respect to the nearest (*N* = 0) Landau level and hence provides an independent measure of the carrier density. We also demonstrate that by extending the existing models^11^,^12^,^16^ for the field dependent charge transfer under the framework of broadened Landau levels, together with our proposed expression for the field dependent carrier density, we obtain a better understanding of the charge transfer process and its effects on the breakdown of the quantum Hall effect in epitaxial graphene grown on SiC.

## Results

### Breakdown of the quantum Hall effect in ultra-low carrier density epitaxial graphene

**Figure 1:**
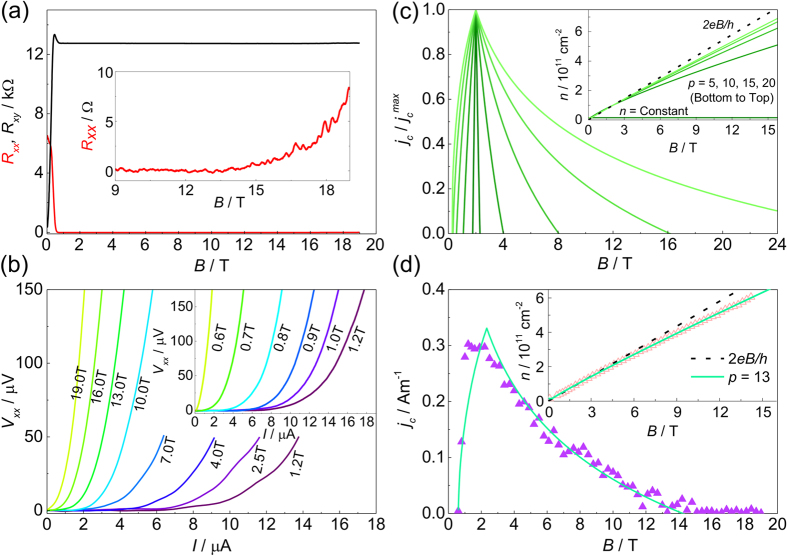
Quantum Hall effect in ultra-low carrier density epitaxial graphene. (**a**) Continuous field measurements of the quantum Hall effect at 2 K using a constant measurement current of 1 *μ*A in an epitaxial graphene on SiC sample with the zero field carrier density reduced to 1.5 × 10^10^ cm^2^ by corona discharge at room temperature. Inset: an expanded plot of *R*_*xx*_ towards the end of the plateau. (**b**) *I* − *V*_*xx*_ traces indicating the onset of the quantum Hall breakdown for the high magnetic field side of the plateau and inset, for the low field side. (**c**) Theoretical dependence of the breakdown current for the *ν* = 2 quantum Hall plateau using equations (1) and (6) with *B*_0_ = 0.3 T and *B*_*c*_ = 2.0 T. A set of dependences are shown with the power, *p*, chosen as 5, 10, 15, and 20. The traditional triangular dependence of breakdown current on magnetic field for constant *n* becomes stretched and asymmetric as the carrier density tends towards an almost linear dependence on magnetic field. The inset shows the corresponding field dependent carrier densities. (**d**) Magnetic field dependence of the breakdown current (purple triangles) for the *ν* = 2 plateau and a best fit (solid line) using equations (1) and (6) with the power *p* = 13. The inset shows the corresponding magnetic field dependent carrier density.

We begin with the sample having its lowest density, where the zero field carrier density is 1.5 × 10^10^ cm^−2^, as determined from the low field Hall coefficient 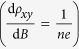. In this state the *ν* = 2 quantum Hall state is observed to begin well below 1 T and the longitudinal resistivity, *R*_*xx*_ is undetectably small from 0.7 T up to 14 T, as shown in Fig. 1(a). Due to its topological origin the quantum Hall state is comparatively robust, in particular to the presence of impurities^22^. However, at high temperatures and high current densities dissipation is introduced into the system and the quantum Hall state breaks down. Above a critical current density *j*_*c*_ = *I*_*c*_/*W* a sudden onset of longitudinal resistance indicates the breakdown of the quantum Hall effect. The value of *j*_*c*_ is defined as the current density above which *V*_*xx*_ > *V*_*c*_, where the critical voltage *V*_*c*_ is determined by the noise limit of the measurement. It can be extracted from the *I* − *V*_*xx*_ characteristics in the quantum Hall state, as shown in Fig. 1(b), where *V*_*c*_ = 0.5 *μ*V. By measuring the breakdown current as a function of magnetic field throughout the *ν* = 2 plateau we are able to extract a wealth of data, in particular about the magnetic field dependent carrier density.

In traditional semiconductor quantum Hall systems, where the carrier density is constant, the breakdown current has been shown to have a triangular dependence on both filling factor and magnetic field, where a sharp peak is observed in *j*_*c*_ centred exactly at an integer filling factor (e.g. *ν* = 2) falling linearly to zero at the edges of the plateau (approximately *ν* = 2 ± *δν*)^23^,^24^,^25^, so that,


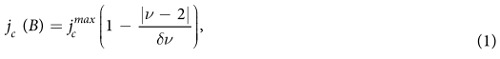


where 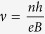 and *δν* is typically in the range 0.2 < *δν* < 0.3^23^,^24^,^25^. As the Fermi energy steadily moves away from the integer filling factor at higher or lower magnetic fields the extended states of the *N* = 0 or *N* = 1 Landau level become energetically easier to access, dissipation occurs at progressively lower measurement currents until *j*_*c*_ decreases to zero. In order to explain the extremely wide plateaus observed in graphene both here and previously^3^,^12^,^16^ it has been assumed that there is a strongly magnetic field dependent carrier density. As a result the occupancy of the *ν* = 2 state changes only slowly with magnetic field thus creating an asymmetric and very broad *j*_*c*_ profile along the plateau (Fig. 1(d)).

### Determining the field dependence of *E*_*F*_

**Figure 2:**
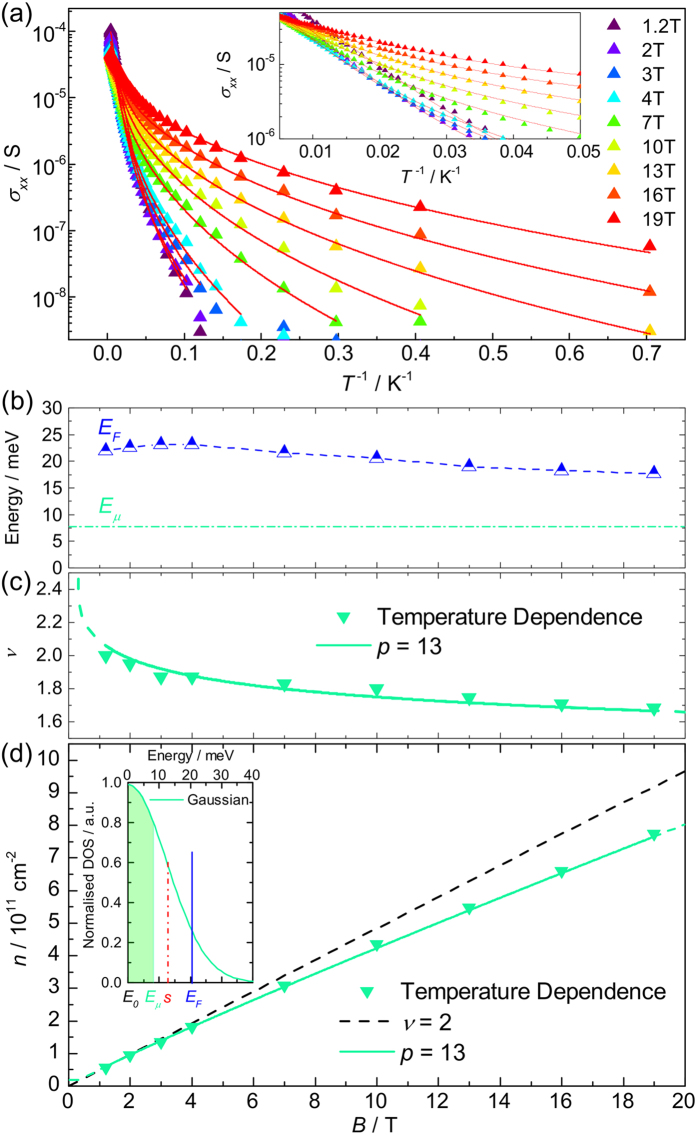
Temperature dependent magneto-conductivity. (**a**) Longitudinal conductivity as a function of temperature from 1.4 to 200 K at various magnetic fields. Red solid lines are fits as described in the text. Inset: high temperature regime from 20 to 200 K. (**b**) *E*_*F*_ and *E*_*μ*_ as a function of magnetic field. (**c**,**d**) Magnetic field dependence of the filling factor *ν* and the carrier density *n* extracted from the temperature dependence analysis (green triangles), and best fits (solid lines) with their extrapolation (dashed lines) using equation (6) with the power *p* = 13. Inset: the Gaussian DOS used in the temperature dependence analysis with characteristic energy levels marked.

In order to produce a quantitative measurement of the magnetic field dependent carrier density, we have studied the temperature dependence of the magneto-conductivity (*σ*_*xx*_) of this sample. The longitudinal conductivities as functions of inverse temperature from 1.4 to 200 K for a series of magnetic fields are shown in Fig. 2(a). The inset shows the relatively high temperature regime from 20 to 200 K where log *σ*_*xx*_ is linear with 1/*T*. In contrast, the non-linearity of the log *σ*_*xx*_ − 1/*T* plot at low temperatures shows the importance of variable range hopping (VRH) which causes significant deviations from the conventional Arrhenius activation behaviour. Excellent fits to the total longitudinal conductivity can be achieved however by combining thermal activation between extended states in adjacent Landau levels and VRH. The total conductivity is calculated as,


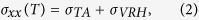


where *σ*_*TA*_ and *σ*_*VRH*_ are due to the thermal activation and variable range hopping, respectively. The most significant outcome of these fittings is the magnetic field dependence of the Fermi energy (*E*_*F*_) which comes mainly from *σ*_*TA*_, dominant at high temperatures. No significant difference in *E*_*F*_ is found by using either the Mott^26^ or Efros-Shklovskii^27^ VRH model for the low-temperature conductivity, and in the subsequent analysis we show results using Mott VRH in two-dimensions^26^, i.e.,


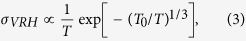


where the characteristic temperature *T*_0_ is a fitting parameter. In terms of *σ*_*TA*_, obtaining a simple analytic formula can be difficult, especially when characteristics of Landau level broadening are taken into account^28^,^29^,^30^. Overall we fit the conductivity using a Gaussian density of states (DOS) function as calculated from a path-integral method^31^,


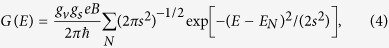


where *g*_*v*_ = *g*_*s*_ = 2 are the valley and spin degeneracies, *E*_*N*_ is the energy of the *N*th Landau level, and the standard deviation *s* represents the Gaussian broadening of a Landau level. In our case the thermal energy is small compared with the Landau level separation and the only contributions are from the extended states within the mobility edges (*E*_*N*_ ± *E*_*μ*_) at the centres of the *N* = 0 and *N* = 1 Landau levels. Since the Fermi level *E*_*F*_ is also well within the region of localised states, from the Kubo formula^30^ we can then write,


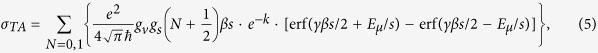


where *k* = *γβ*(*E*_*N*_ − *E*_*F*_) − (*βs*/2)^2^, 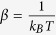, and 
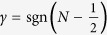
.

Employing the above formulation, the temperature-dependent magneto-conductivities are very well fitted across a broad temperature range from 1.4 up to 200 K, as shown in Fig. 2(a). To avoid overfitting, we assume the DOS of the *N* = 0 and *N* = 1 Landau levels have fixed Gaussian broadening, i.e., *s* and *E*_*μ*_ are constants independent of magnetic field and temperature. The value of *s* is chosen to be ~12 meV in order to match the energy scale of the low-field Gaussian random disorder potentials in these systems^32^. *E*_*μ*_ is determined to be 7.8 meV (|Δ*ν*| = 0.93, i.e., almost 50% of the total states of each Landau level are extended) by fitting the data for *B* = 1.2 T, where *E*_*F*_ is known to be exactly at the mid-point between the two Landau levels since at elevated temperatures the minimum in conductivity and *R*_*xx*_ is observed at this field. We also note that the dimensionless parameter 
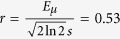
 in our sample is consistent with the value 0.5, which has been used to interpret similar Gaussian Landau level broadening and extended states widths from transport spectroscopy measurements in epitaxial graphene^33^. As the magnetic field increases, Fig. 2(b) shows that *E*_*F*_ is a slowly decreasing quantity which approaches the mobility edge *E*_*μ*_ gradually. This suggests that the magnetic field dependent charge transfer rate is slightly lower than the increase in the total Landau level DOS. Figure 2(c,d) show the filling factor and the corresponding carrier density as functions of magnetic field, i.e. 
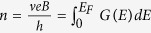
, where *E*_*F*_ as a function of magnetic field is given by Fig. 2(b) and *G*(*E*) is given by equation (4).

In order to provide a quantitative relation to describe the charge transfer which can be compared to the magnetic field dependence of the breakdown current, we introduce the following phenomenological expression for the field dependent carrier density,


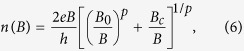


where *p*, *B*_0_ and *B*_*c*_ are fitting parameters. This expression is chosen as, for large values of *p*, it rapidly swaps over from a constant carrier density *n*_0_ at low field, where 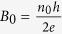 corresponding to the magnetic field where *ν* = 2 would occur if there were no charge transfer at this low field carrier density, to a slightly sublinear increase with field 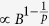, where *B*_*c*_ is the magnetic field corresponding to 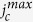 where *ν* = 2 including the additional charge transfer. The carrier density and occupancy predicted by this equation for this sample are shown in Fig. 2(c,d) and can be seen to provide an excellent description of the field dependence of both parameters when *p* = 13. Using the analytical form for *n* given by equation (6) we can now combine this with the expected form of the occupancy dependence of the quantum Hall break down current given by equation (1) to give a prediction for the field dependence of the breakdown current. A series of such breakdown predictions are shown in Fig. 1(c) for *p* = 5, 10, 15 and 20, which demonstrate the appearance of a strong asymmetry as a function of field and a very considerable broadening of the breakdown current field dependence. Figure 1(d) then compares the field dependence of *j*_*c*_ to the data deduced from Fig. 1(b) with the optimum fitting parameter of *p* = 13 which gives an excellent description of the data. We use *p* = 13 in all subsequent fitting. The magnetic field at which *j*_*c*_ goes to zero (14 T) corresponds to an occupancy as deduced from the temperature dependence of *σ*_*xx*_ of 1.74 (Fig. 2(c)) so that we take *δν* = 0.26 in equation (1), which is in good agreement with other measurements in both graphene and other systems^23^,^24^,^25^. In summary therefore we can say that the measurement of the field dependence of *j*_*c*_ is well described by fitting with equations (1) and (6) and inversion of the fitting process also allows us to use the measurement of *j*_*c*_ as an independent measurement of the field dependent occupancy and hence of the field dependent carrier density, as shown in the inset to Fig. 1(d). We will later show that equation (6) also agrees well, in the vicinity of the *ν* = 2 plateau, with full numerical simulations as described below.

### Pulsed field measurements of a 50 T wide *ν* = 2 plateau

**Figure 3:**
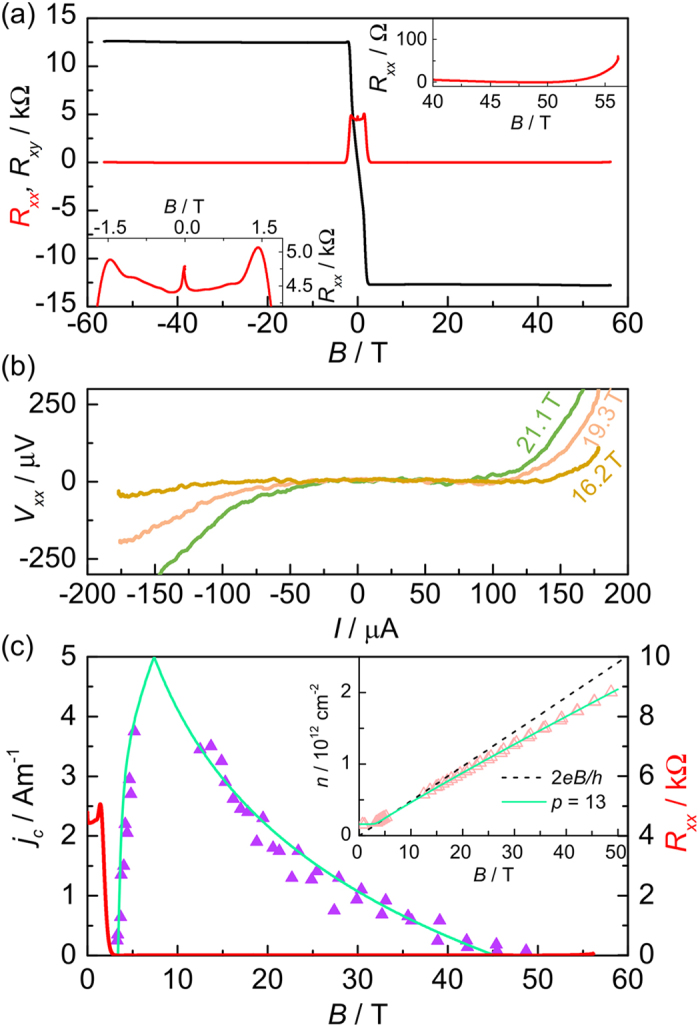
Giant quantum Hall plateaus. Pulsed magnetic field measurements of the quantum Hall effect in epitaxial graphene on SiC at *T* = 2 K as observed in the longitudinal resistance (*R*_*xx*_, red) and Hall resistance (*R*_*xy*_, black) showing an exceptionally wide (~50 T) *ν* = 2 plateau. Upper inset: The end of the plateau at *B* > 50 T. Lower inset: *R*_*xx*_ at low magnetic fields showing clear Shubnikov-de Haas oscillations and weak localisation. (**b**) Examples of high speed *I* − *V*_*xx*_ traces taken during a single pulsed magnetic field measurement, showing that clear quantum Hall breakdown behaviour is observed. (**c**) Magnetic field dependence of the breakdown current (purple triangles) for the *ν* = 2 plateau and a best fit (solid line) using equations (1) and (6) with the power *p* = 13. The red line shows the longitudinal resistivity and the inset shows the corresponding magnetic field dependent carrier density.

Using the above method we now use *j*_*c*_ to probe the carrier density up to much higher magnetic fields. Figure 3(a) shows the magnetic field dependence of the the longitudinal and Hall resistance, *R*_*xx*_ and *R*_*xy*_ respectively, measured using a constant (DC) current of 3 *μ*A in a 57 T long pulse magnet. At both positive and negative magnetic fields the magnetotransport is dominated by the extended *ν* = 2 plateau. Using the low field Hall coefficient we extract an initial carrier density of 1.5 × 10^11^ cm^−2^. Also at low magnetic fields we observe a clear weak localisation peak in the resistivity^34^, and even signatures of Shubnikov-de Haas oscillations for *ν* = 6 and *ν* = 10 (Fig. 3(a), lower inset). This shows that even with a total pulsed magnetic field measurement duration of about 300 ms the quality and accuracy of the data remains very high. The dissipationless *ν* = 2 state begins at 3 T and is accompanied by a well quantised Hall resistance. Eventually at above *B* = 50 T, *R*_*xx*_ begins to increase as the Fermi level approaches the *N* = 0 Landau level and the extended states begin to be thermally populated. In order for the zero resistivity state to exist over this enormous magnetic field range this implies that the occupancy remains close to *ν* = 2 due to a rapidly increasing carrier density as previously proposed. To get a greater insight into the magnetic field dependent carrier density we investigate the breakdown of the quantum Hall state at *ν* = 2 by studying the increase of *R*_*xx*_ at high measurement currents. To determine the value of *I*_*c*_ as a function of magnetic field during a magnetic field pulse, we measure a set of *I* − *V*_*xx*_ traces using a 107 Hz alternating current source and digital oscilloscope with a sampling rate set at 1 MHz. Example traces are shown in Fig. 3(b). These traces are measured from both a rapid sinusoidal upsweep (50 ms) and a slower downsweep with an exponential decay constant of 100 ms without showing any visible hysteresis. We therefore estimate the time constant of the charge transfer process to be a few milliseconds or less. From this we can extract *I*_*c*_ along the plateau, as shown in Fig. 3(d). The values extracted using this method compare well to those taken in the lower field range using steady-field measurements on the same sample.

As before we now use equations (1) and (6) to fit the field dependence of *j*_*c*_, varying only the parameters *B*_0_ and *B*_*c*_ as shown in Fig. 3(c). The fit is again excellent and when inverted to provide a measure of the carrier density from *j*_*c*_, the density is found to increase from a zero field value of 1.5 × 10^11^ cm^−2^, by over an order of magnitude to 2 × 10^12^ cm^−2^ at *B* = 50 T (Fig. 3(c) inset).

### Overview of samples

**Figure 4:**
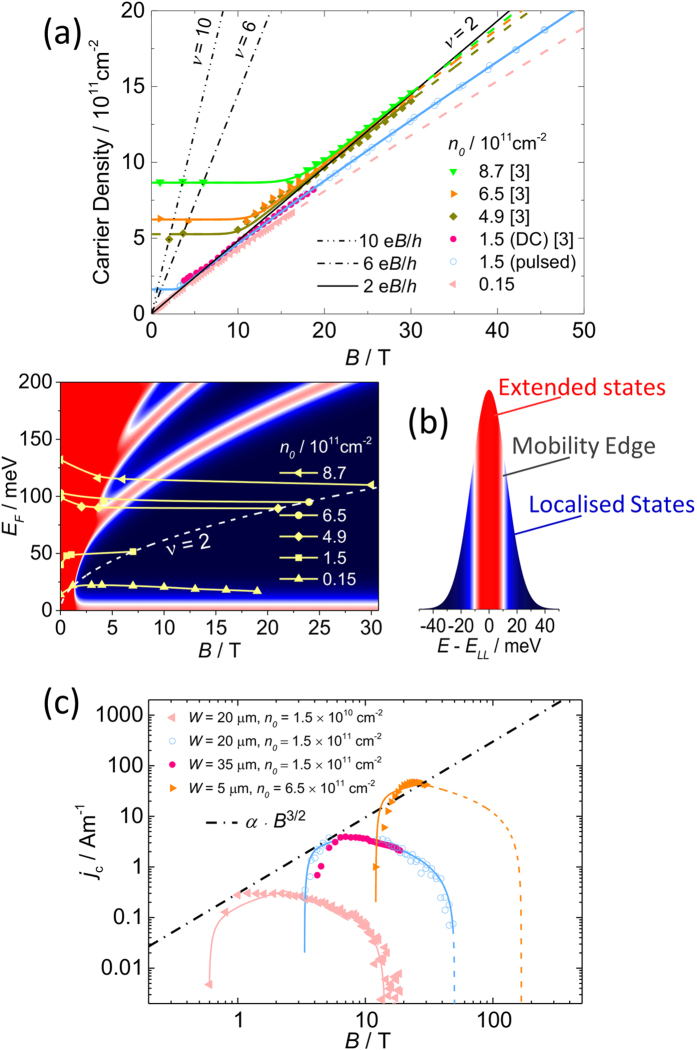
Zero field carrier density dependence of charge transfer. (**a**) Comparison of the carrier density deduced from the quantum Hall breakdown current with the values fitted with equation (6) for several measurements on samples with different zero-field carrier densities. (**b**) The extracted magnetic field dependence of the Fermi energy in these samples based on the magnetic field assignments for *ν* = 4*n* + 2. (**c**) Magnetic field dependence of *j*_*c*_, showing that the peak breakdown current follows *B*^3/2^ as observed in other quantum Hall systems. The solid lines show the predicted values from the fitting from equations (1) and (6).

Using these results in combination with those previously published^3^ we may now make a complete comparison of the magnetic field dependent carrier density of several samples with *n*_0_ spanning almost three orders of magnitude. Figure 4(a) shows the fitted carrier density using equations (1) and (6) as a function of magnetic field from six sets of measurements. The carrier density in all of the samples show a strong magnetic field dependence in the *ν* = 2 regime. Additional measurements of the carrier density at low magnetic fields, corresponding to the low field Hall coefficient and the magnetic field at which the *ν* = 6 and 10 resistivity minima occur, show that carrier density remains almost constant at high filling factors. Finally, by using data points where the occupancy is known accurately such as at the resistivity minima for *ν* = 6 and 10 and the field at which *ν* = 2 peak breakdown occurs, we may estimate the chemical potential to be midway between Landau levels. This gives us an estimate of the chemical potential as a function of magnetic field in all of the samples studied, in addition to the values extracted from the temperature dependence described above, which is plotted in Fig. 4(b) relative to the energy of the Landau levels. In all cases the chemical potential is falling very slowly with increasing magnetic field. Together with the field dependence of the carrier density these results suggest the presence of charge reservoirs in close proximity to the graphene with exceptionally high densities of states. Using the field dependence of *E*_*F*_ and *n* in Figs 2 and 4, we can estimate these to be in the order of 10^14^ cm^−2^ eV^−1^ which is over an order of magnitude larger than suggested in initial reports^12^. Such a large density of states could arise from defects within the first few Si-C layers which may be created alongside the Si-sublimation during the graphene growth, and also from the charge traps in the interface between graphene and the top gate materials.

These new data, combined with those published previously now also allow us to investigate the magnetic field dependence of the peak values of *j*_*c*_, as shown in Fig. 4(c). We see that over a wide range of magnetic fields *j*_*c*_ ∝ *B*^3/2^, which has been observed previously in several studies in GaAs^24^,^35^,^36^ and graphene^3^. In addition by using the functional form of the carrier density given by equation (6) we can predict the highest field limit at which the quantum Hall state should still exist 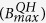 for the current batch of samples as,


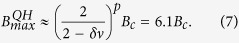


### Modelling the magnetic field dependent charge transfer process

To get more insight into the magnetic field dependent charge transfer as discussed above and its effects on the breakdown of the quantum Hall effect, we now propose a model which takes account of the interface states as charge reservoirs and the effects of quantum capacitances from each part of the system. We note that all our above analysis is based on the picture of broadened Landau levels, instead of the unbroadened *δ*-function Landau levels which are assumed in the existing theoretical models^12^,^16^. We therefore view our model as an extension of these models to a framework of broadened Landau levels.

**Figure 5:**
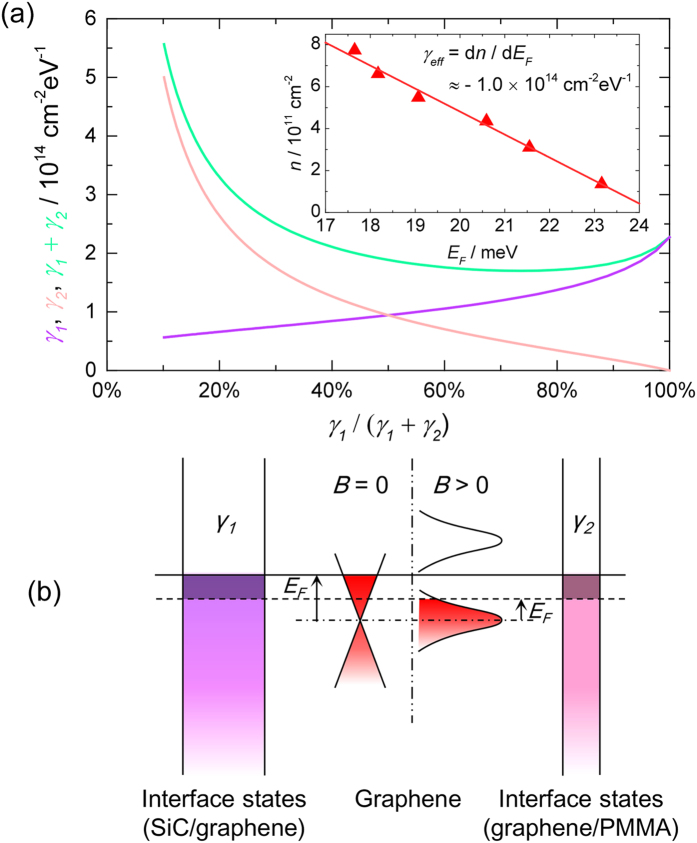
Determining the density of interface states. (**a**) Relation between the total density of interface states in the two charge reservoirs (*γ*_1_ + *γ*_2_, green) and the individual component for SiC (*γ*_1_, purple) and PMMA (*γ*_2_, pink). Inset: carrier density as a function of Fermi level in our epitaxial graphene. (**b**) Schematic energy diagram of graphene at fixed gate voltage between two charge reservoirs with DOS *γ*_1_ and *γ*_2_, at *B* = 0 and *B* > 0. Shaded areas in the left and right reservoirs represent the additional electrons transferred into graphene due to magnetic field.

Given that all our epitaxial graphene samples are static gated with PMMA as a gate insulator on top of the graphene, we have adopted equation (A12) from ref. 16. At equilibrium with fixed gate voltage, we can write,


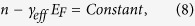


and,


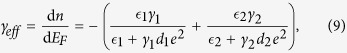


where *ϵ*_1(2)_, *γ*_1(2)_, and *d*_1(2)_ are the absolute permittivity, the assumed constant DOS of surface donor states, and the distance between graphene and the donor states for the SiC/graphene (graphene/PMMA) interface, respectively (Fig. 5(b)). It is worth pointing out that in equation (8), no assumption about the details of the DOS in graphene is required. Therefore, it is valid for both the equilibrium at zero field and the equilibria at high fields where the DOS of each Landau level is broadened. As the magnetic field increases, *γ*_*eff*_ is effectively the number of charges transferred into graphene per unit change of the Fermi energy, and it is a negative quantity as indicated in equation (9) since the carrier density is increasing while the Fermi level is gradually falling to the mobility edge (Figs 2(b) and 4(b)) and eventually to zero.

Figure 5(a) inset shows the carrier density as a function of the Fermi energy from the temperature dependence measurement (Fig. 2(b,d)). It is well fitted with a straight line with its slope indicating *γ*_*eff*_ ≈ −1.0 × 10^14^ cm^−2^ eV^−1^ in our sample. Using (*ϵ*_1_, *ϵ*_2_) = (9.7*ϵ*_0_, 3.5*ϵ*_0_) (*ϵ*_0_ = vacuum permittivity), *d*_1_ = *d*_2_ = 0.3 nm, we plot the relation between *γ*_1_ and *γ*_2_ in Fig. 5(a) based on equation (9). It is observed that when the density of surface states in SiC (*γ*_1_) decreases from 30% to 0% of the total DOS in the two charge reservoirs, *γ*_2_ will rapidly increase to infinity. It is not physical to have such a large DOS in the PMMA interface (*γ*_2_), so it is unlikely that 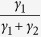 will take a value below 30%. On the other hand, *γ*_1_ + *γ*_2_ remains roughly constant over a large range, 
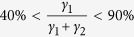
. We can therefore estimate the DOS in the reservoirs to be *γ*_1_ ~ 0.8 − 1.7 × 10^14^ cm^−2^ eV^−1^, *γ*_2_ ~ 0.3 − 1.3 × 10^14^ cm^−2^ eV^−1^, with *γ*_1_ + *γ*_2_ ~ 2 × 10^14^ cm^−2^ eV^−1^. Our estimation suggests that the surface states in SiC play a more significant role in the charge transfer process. The surface states responsible are closely related to the large number of atomic defects (vacancies and adatoms) predominantly located in the first underlying atomic layer, which consists of a non-conductive surface reconstruction of carbon atoms covalently bonded to the SiC which is formed during the growth process^12^. The existence of such surface defects with visually similar densities within this interfacial layer has been extensively reported from photo-emission and scanning tunneling microscopy studies on various surface reconstructions^37^,^38^,^39^,^40^,^41^,^42^,^43^,^44^,^45^. These experiments have shown the existence of surface states distributed over an energy range of 0.5–1.5 eV in the region close to *E*_*F*_^37^,^40^,^43^,^44^,^45^ and have determined defect densities of order 2–4%. Assuming that one surface state originates from each defect site and the surface states are distributed in a typical energy range of 1 eV^42^ within the bandgap, then using an atomic density ~3.7 × 10^15^ cm^−2^ for the reconstructed layer leads to DOS in the range of 0.8 − 1.5 × 10^14^ cm^−2^ eV^−1^. Despite the variations in experimental conditions, the reported values are consistent with our estimate for *γ*_1_. For the graphene/PMMA interface, we attribute the less significant but still relatively high *γ*_2_ to the increased number of charge traps introduced by the intensive UV treatment^20^,^46^, and/or the high electric field involved in the corona gating process^21^,^47^.

**Figure 6:**
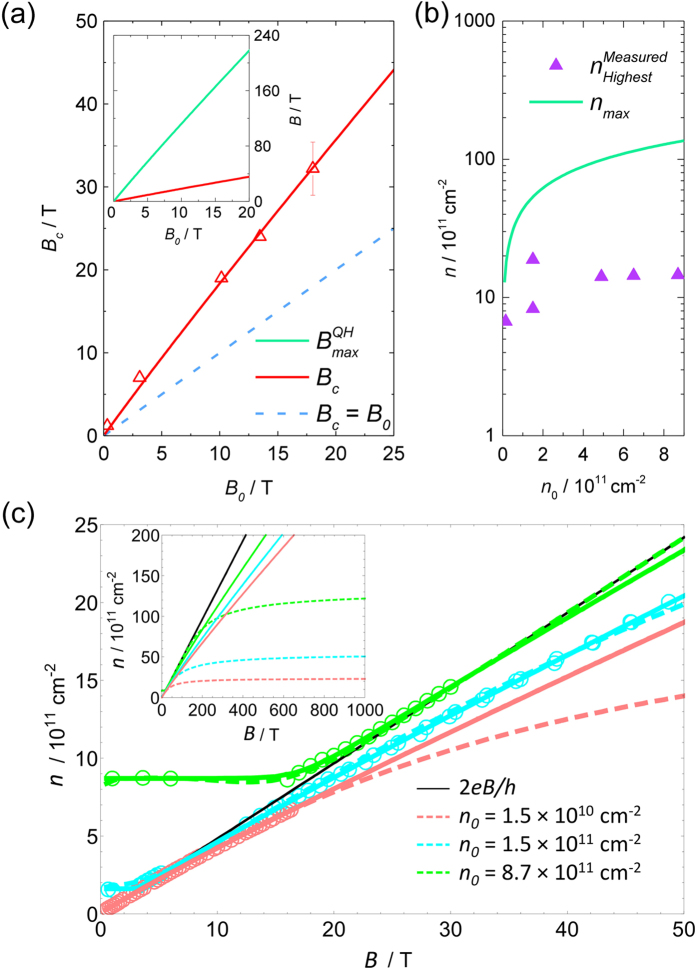
Numerical simulations of charge transfer. (**a**) *B*_*c*_ as a function of *B*_0_, including our experimental data (red triangles), the prediction using equation (10) (red solid line), and the case without charge transfer (blue dashed line). Inset: 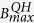 (green solid line) and *B*_*c*_ (red solid line) as functions of *B*_0_. (**b**) Comparisons between *n*_*max*_ (green line) as a function of *n*_0_ and 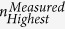 (purple triangles) for our samples. (**c**) Simulations of complete *n*(*B*) dependences given by our charge transfer model (dashed lines), compared with the experimental data (open circles) and equation (6) (pink, cyan, green solid lines). Inset: Same comparisons up to extremely high magnetic fields.

Another important consequence from our model is that we can deduce *B*_*c*_ (the magnetic field corresponding to the occupancy *ν* = 2 where the minimum *R*_*xx*_ and the maximum *j*_*c*_ occur) as a function of the zero field carrier density. Based on equation (8), we have,


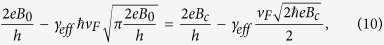


where *v*_*F*_ is the Fermi velocity. The left hand side and the right hand side of the equation represent the charge equilibrium at *B* = 0 and *B* = *B*_*c*_, respectively, with *E*_*F*_ being 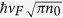 at *B* = 0, and at the mid-point between the *N* = 0 and *N* = 1 Landau levels 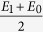 at *B*_*c*_. The solution to equation (10) is plotted against *B*_0_ (red lines) in Fig. 6(a), which is in excellent agreement with our experimental data (red triangles). Also shown (blue dashed line) is *B*_*c*_ = *B*_0_, which represents the case of a constant carrier density without the field dependent charge transfer. Combining equations (7) and (10), we can also predict the magnetic field, 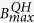, at which the end of the *ν* = 2 plateau occurs, using *B*_0_ as shown in the Fig. 6(a) inset.

Finally, we calculate the maximum carrier density *n*_*max*_ that can be transferred into graphene, corresponding to the limit *B* → ∞ and *E*_*F*_ → 0 when other high magnetic field effects (such as the fractional quantum Hall effect) are neglected. From equation (8), we have,


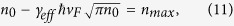


or,


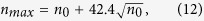


where *n*_*max*_ and *n*_0_ are both in units of 10^11^ cm^−2^. This relation is shown in Fig. 6(b). *n*_*max*_ represents the upper limit to the possible validity of equation (6) which occurs when the Fermi energy becomes pinned to the Dirac point as *B* → ∞. In practice, the highest carrier densities reached in our measurements, 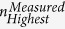, as shown in Fig. 6(b), are much less than *n*_*max*_ due to the finite magnetic field strength we could apply. Therefore, in this regime, equation (6) still provides an accurate description for *n*(*B*).

Hence, with our model using a realistic framework of broadened Landau levels, we have been able to relate and accurately predict some of the most important characteristics (*B*_*c*_, 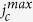, 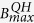, *n*_*max*_) of the breakdown of the quantum Hall effect and the field dependent charge transfer, using just the zero field carrier density (*n*_0_ or *B*_0_). These results can thus provide realistic references for the optimum operating conditions for a quantum Hall resistance standard using epitaxial graphene. We emphasise that our model can ultimately provide a complete *n*(*B*) dependence, if the exact details of the Landau level broadening are known. Such dependences are simulated in Fig. 6(c), for three samples with each *n*_0_ listed, by numerically solving the equation set,


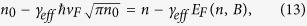



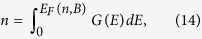


where *G*(*E*) is the DOS of Landau levels. In this simulation, we continue to assume Gaussian Landau level broadening, such that *G*(*E*) is given by equation (4), the same as in the above analysis of the temperature dependent conductivity. We have also used the same standard deviation *s* = 12 meV of the Gaussian broadening for the same sample (*n*_0_ = 1.5 × 10^10^ cm^−2^) on which the temperature dependent measurements were made. For the samples with *n*_0_ = 1.5 and 8.7 × 10^11^ cm^−2^ in which *s* is unknown, our model suggests values of about 25 and 30 meV, respectively, both well within the normal range among similar samples^32^. As shown in Fig. 6(c), throughout the measurement range for each sample (i.e. 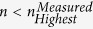), excellent agreement is observed between the experimental data (open circles), the *n*(*B*) dependences given by our charge transfer model (dashed lines), and the those given by equation (6) with *p* = 13 (solid lines). At high enough magnetic fields, the inset to Fig. 6(c) shows that our model deviates from equation (6) and predicts an upper limit to the number of electrons that can be transferred into graphene, corresponding to *n*_*max*_ as given by equation (12) and shown in Fig. 6(b).

## Discussion

In summary, we have studied the breakdown and the temperature dependence of the quantum Hall effect using high magnetic fields and very low density epitaxial graphene grown on SiC. Our new measurements show that the full width of the *v* = 2 plateau can be observed in this system for low enough starting densities, *n*_0_, and demonstrate the very large extent of the magnetic field dependent carrier density in epitaxial graphene. We have shown that the quantum Hall effect breakdown current can be used to accurately measure this increase in carrier density with field, which is found to be over an order of magnitude in some cases. Using the models that we propose, we have been able to accurately describe and predict some of the most important features of the field dependent charge transfer process and its effects on the quantum Hall breakdown. Our models and results are widely applicable towards a broader and deeper understanding of the high magnetic field transport properties of graphene and are crucial for engineering epitaxial graphene devices for applications such as quantum Hall metrology.

## Methods

The devices studied were prepared from epitaxially grown graphene on the Si-terminated face of SiC. Each device was patterned using electron beam lithography and oxygen plasma etching into an eight-leg Hall bar geometry with a width *W* = 20 *μ*m and length *L* = 20 *μ*m. Electrical contacting was made using large area electron beam evaporated Ti-Au. A non-volatile dual-polymer gating technique using PMMA/MMA and ZEP520A was applied to tune the carrier density in the epitaxial graphene using a combination of UV illumination^20^ and corona discharge^21^ at room temperature. Pulsed magnetic fields of up to 57 T were provided by a 19 kV long pulse magnet at the LNCMI-Toulouse, and continuous field measurements were taken in a superconducting solenoid magnet. Measurements were taken at a fixed temperature of *T* = 2 K unless otherwise stated.

## Additional Information

**How to cite this article**: Alexander-Webber, J. A. *et al.* Giant quantum Hall plateaus generated by charge transfer in epitaxial graphene. *Sci. Rep.* **6**, 30296; doi: 10.1038/srep30296 (2016).
